# Comparison of outcomes after 3-month methadone maintenance treatment between heroin users with and without HIV infection: a 3-month follow-up study

**DOI:** 10.1186/s12954-015-0047-0

**Published:** 2015-05-08

**Authors:** Peng-Wei Wang, Huang-Chi Lin, Chia-Nan Yen, Yi-Chun Yeh, Chih-Yao Hsu, Kuan-Sheng Chung, Hsun-Cheng Chang, Hung-Chi Wu, Cheng-Fang Yen

**Affiliations:** Department of Psychiatry, Kaohsiung Medical University Hospital, Kaohsiung, Taiwan; Department of Psychiatry, Faculty of Medicine and Graduate Institute of Medicine, College of Medicine, Kaohsiung Medical University, 100 Tzyou 1st Road, Kaohsiung, 807 Taiwan; Department of Psychiatry, Tainan Hospital, Department of Health, Executive Yuan, Tainan, Taiwan; Department of Addiction Science, Kaohsiung Municipal Kai-Syuan Psychiatric Hospital, No. 130, Kaisyuan 2nd Rd., Lingya Dist Kaohsiung City, 80276 Taiwan; Department of Community Psychiatry, Kaohsiung Municipal Kai-Syuan Psychiatric Hospital, No. 130, Kaisyuan 2nd Rd., Lingya Dist Kaohsiung City, 80276 Taiwan

**Keywords:** Heroin, Methadone, HIV, Depression, Quality of life

## Abstract

**Background:**

The aim of this study was to compare the changes in primary (heroin use-related) and secondary (depressive symptoms and quality of life, QOL) outcome indicators of 3-month methadone maintenance treatment (MMT) between heroin users with and without HIV infection.

**Methods:**

A total of 242 intravenous heroin-dependent individuals (30 with and 212 without HIV infection) receiving MMT were recruited. Primary (severity of heroin dependence, harm caused by heroin use and current heroin use) and secondary (depressive symptoms and QOL) outcome indicators were determined before and after receiving 3-month MMT. Changes in primary and secondary outcome indicators between the two groups were compared using mixed-model analysis.

**Results:**

Heroin users both with and without HIV infection showed significant improvement in three primary outcome indicators after 3-month MMT, and there was no difference in the changes of these primary outcome indicators between the two groups. However, improvements in depressive symptoms and the physical domain of QOL among HIV-infected heroin users were poorer than in those without HIV infection.

**Conclusions:**

The results of this study indicated that heroin users with HIV infection did improve in the primary but not the secondary outcomes after 3-month MMT.

## Introduction

Heroin dependence is associated with poor physical and mental health, unemployment and criminal activity and has a chronic, relapsing course [1,2]. A recent study in Taiwan found that the economic cost of heroin dependence is huge, equal to 1.07 times the average gross domestic product per capita in Taiwan [3]. Another major problem associated with heroin use is the spread of blood-borne infections, such as human immunodeficiency virus (HIV), among injecting drug users (IDUs). In Taiwan, it was reported that only 2.1% of HIV-positive individuals were IDUs in 2003 [4]. An outbreak of HIV type 1 infection in Taiwan during 2003–2008 was caused by a new strain of HIV CRF07_BC [5]. It has been estimated that IDUs contributed 38.5% of accumulated HIV cases by the end of 2007 [6]. To control the HIV epidemic among IDUs, harm reduction programs were introduced in Taiwan for heroin users both with and without HIV infection in 2005 [5]. Methadone maintenance treatment (MMT) is a core component of the harm reduction programs that have been recommended. MMT has been available for heroin-dependent individuals since the 1960s [7] and has been demonstrated to be a cost-effective intervention in terms of reducing heroin use, criminal behavior, risk of injection-related blood-borne diseases and premature mortality among heroin users [8]. After the implementation of MMT, the annual percentage of new HIV infections from injected drug use decreased dramatically [9], which further supports the benefits of MMT in the control of blood-borne infections among IDUs.

An important issue that requires further study is whether the treatment outcomes of MMT are the same between heroin users with and without HIV infection. A cohort study showed that HIV-infected individuals have higher rates of depression and heroin use disorder than those not infected with HIV [10]. In a study of crack users, HIV-positive users were more likely to be homeless and unemployed during treatment than those negative for HIV [11]. Compared with HIV-negative heroin users, heroin use has an immunosuppressive effect on the central nervous system [12]. Heroin users with HIV infection have a higher rate of comorbid infection with hepatitis C virus than HIV-negative heroin users [13]. These study results show that HIV-infected heroin users are a unique subgroup among heroin users. This highlights the importance of examining whether there are differences in the treatment outcomes of MMT between heroin users with and without HIV infection.

Whereas the severity of heroin dependence and harm caused by heroin use and the occurrence of heroin use are important outcome indicators of MMT, the levels of depressive symptoms and quality of life (QOL) are also important indicators. A 12-month follow-up study among heroin users showed that more severe depressive symptoms at baseline can predict more injection-related health problems and heroin use and less heroin abstinence 12 months later [14]. Therefore, effective treatment of depressive symptoms in heroin users is an important issue [15]. Regarding the effects of MMT on depressive symptoms, previous studies have reported that MMT can lessen the severity of depressive symptoms among heroin users [16,17]. However, whether there is a different effect of MMT on improving depressive symptoms between heroin users with and without HIV infection remains unknown. Depressive symptoms are common among people with HIV infection [15]. In a nationally representative probability sample of adults receiving care for HIV infection, there is a 5–7% higher 12-month prevalence rate of depressive disorders in individuals with HIV infection than in the general population [18]. Further study is needed to examine whether there are differences in the effect of MMT on improving depressive symptoms between heroin users with and without HIV infection.

QOL has been defined by the World Health Organization as a term of “individuals’ perceptions of their position in life in the context of the culture and value systems in which they live and in relation to their goals, standards, expectations and concerns” [19]. Research has found that heroin users have a poorer QOL than those who do not use heroin [20]. Meanwhile, individuals with HIV infection have to deal with a series of problems, such as physical and psychological discomfort, economic stress, social stigma and the fear of death, and thus QOL can be compromised by HIV infection [21]. Whether there is a different effect of MMT on improving the levels of QOL between heroin users with and without HIV infection requires further study.

This study aimed to examine the changes in primary (severity of heroin dependence, harm caused by heroin use and current heroin use) and secondary (depressive symptoms and QOL) outcome indicators during 3-month MMT in heroin users with and without HIV infection. The group differences in the changes of these outcome indicators were also examined.

## Methods

### Research design and participants

This observational study recruited consecutive intravenous heroin users with heroin dependence according to the criteria of the DSM-IV-TR [22] who visited the MMT clinics of three hospitals (two general hospitals and one psychiatric hospital) in southern Taiwan from March 2007 to July 2008. To achieve a desired power higher than 0.8, the minimum sample size was 168 [23,24]. A total of 242 heroin users agreed to participate in this 3-month follow-up study. All participants had not received methadone previously. All participants received an HIV serostatus test by ELISA, and if the screening test was positive, western blot was used as a confirmatory test. If the result was positive, participants were confirmed as HIV-positive. Of 242 participants, 30 were HIV-positive and 212 were HIV-negative. All HIV-positive participants were at the clinical latency stage and were not on any antiretroviral therapy. Regarding methadone dosage, doctors adjusted dosages to adequately reduce craving and opioid withdrawal symptoms in the first month of treatment for both groups. Then participants continued taking their stabilization dose through to the end of this study. They underwent intake interviews to collect baseline data before receiving MMT and were evaluated again after receiving 3-month MMT. At each evaluation, participants were interviewed face to face by research assistants who were blind to the baseline data, to collect the outcomes at the follow-up points. The study protocol was approved by the Institutional Review Board of Kaohsiung Medical University.

### Survey instruments

At each interview, the instruments described below were used to collect data.

#### The Chinese version of the severity of dependence scale (SDS^[Ch]^)

The five-item SDS^[Ch]^ was used to evaluate participants’ severity of heroin use [25,26]. Each item is scored on a four-point scale (scored 0 to 3). The total SDS score ranges from 0 to 15, with higher scores indicating a greater degree of dependence. Cronbach’s alpha of the SDS^[Ch]^ in this study was 0.78.

#### Current heroin use

Participants reported whether they had used heroin before intake and during the last month of the study. Each participant had urine tests at the end of the study. Current heroin use at baseline and at the 3-month follow-up interview was determined by either self-reported use in the last month or by a positive heroin confirmation urine test at clinic visits.

#### Questionnaire for harm of heroin use (Q-HOU)

This seven-item, four-point questionnaire assesses the subjective harmful experiences caused by heroin use during the past 3 months, including those related to physical and mental health, family relationships, interpersonal relationships, legal problems, academic or occupational performance, and economic hardship. For example, participants were asked, “Have you ever experienced physical problems due to heroin use during the past 3 months?” The 1-week test-retest reliability ranged from 0.394 (*p* < 0.05) to 0.742 (*p* < 0.001). Cronbach’s alpha of the Q-HOU in this study was 0.93. Higher total scores on the Q-HOU indicate that individuals are experiencing more severe harm owing to heroin use.

#### Chinese version of the center for epidemiological studies depression scale (CES-D)

The Chinese version of the CES-D has been used to study depressive symptoms in Taiwan for many years [27]. Possible total scores on the CES-D range from 0 to 60, with higher scores indicating more severe depressive symptoms. The internal consistency (Cronbach’s alpha) of the Chinese version of the CES-D in this study was 0.86.

#### Taiwan brief version of the world health organization quality of life instrument (WHOQOL-BREF)

The Taiwan version of the WHOQOL-BREF includes 28 items, consisting of 26 standard items from the original WHOQOL-BREF and two Taiwanese national items [28]. This is a self-administered questionnaire that assesses the levels of QOL in the health, psychological, environment and social relationship domains. The internal consistency coefficients (Cronbach’s alpha) of the Taiwan version of the WHOQOL-BREF ranged from 0.70 to 0.77 for the four domains. The test-retest reliability coefficients with intervals of 2 to 4 weeks ranged from 0.41 to 0.79 at the item/facet level and 0.76 to 0.80 at the domain level (all *p* < 0.01). The content validity coefficients were in the range of 0.53 to 0.78 for item-domain correlations and 0.51 to 0.64 for inter-domain correlations (all *p* < 0.01) [28].

#### Attendance rate of MMT

The attendance rate of MMT was determined by the days on which methadone was taken during the 3-month MMT.

### Statistical analysis

Data analysis was performed using the Statistical Package for the Social Sciences (SPSS) 20.0 software. Baseline characteristics of the participants were compared between participants with and without HIV infection using the *χ*^2^ test and *t*-test. Changes in the severity of heroin dependence, harm caused by heroin use and depressive symptoms and the levels of QOL during the 3-month MMT were examined separately by the paired *t*-test in the two groups of participants. Change in status of current heroin use before and after the 3-month MMT was examined separately by the related-samples McNemar test in the two groups of participants. Meanwhile, we used generalized linear mixed-model analysis to examine group differences in the changes in heroin dependence, harm caused by heroin use, status of current heroin use, depressive symptoms and QOL during 3-month MMT between heroin users with and without HIV infection. Mixed-model analysis is useful in settings where unbalanced measurements are made of the same statistical units [29]. In the mixed-model analysis, changes in the outcome indicators were used as dependent variables, the group variable was used as an independent variable and sex, age, education level and attendance rate of MMT were used as control variables. We used the 0.05 significance level for all inferential statistical procedures.

## Results

Of the 30 participants with HIV infection, five (16.67%) were female and 25 (83.33%) were male. Of the 212 participants without HIV infection, 25 (11.79%) were female and 187 (88.21%) were male. There was no significant sex difference between the two groups. The mean age (standard deviation, SD) was 37.20 years (7.48 years) and 37.73 years (7.29 years) in participants with and without HIV infection, respectively. The mean duration of education (SD) was 9.84 years (2.37 years) and 9.25 years (2.14 years) in participants with and without HIV infection, respectively. There were no significant differences in sex, age and duration of education between participants with and without HIV infection. The severities of heroin dependence, harm from heroin use and depressive symptoms, the levels of QOL, and the proportion rate of current heroin use at baseline in participants both with and without HIV infection are shown in Table 1. There were no significant differences in these indicators at baseline between participants with and without HIV infection.

**Table 1:** **Comparison of treatment outcomes before and after MMT in heroin users with and without HIV infection**

	**Heroin users with**		***p***	**Heroin users without**		***p***
	**HIV infection**			**HIV infection**		
	**Mean (SD) or** ***n*** **(%)**			**Mean (SD) or** ***n*** **(%)**		
	**Baseline**	**3 months after receiving MMT**		**Baseline**	**3 months after receiving MMT**	
Severity of heroin dependence on the SDS^[Ch]^	9.10 (2.89)	5.60 (2.57)	<0.0001^a^	9.27 (2.91)	6.30 (2.36)	<0.0001^a^
Severity of harm caused by heroin use	14.77 (5.29)	7.00 (6.31)	<0.0001^a^	13.69 (4.98)	8.34 (6.31)	<0.0001^a^
Current heroin use	30 (100)	24 (80)	<0.0001^b^	212 (100)	153 (72.17)	<0.0001^b^
Severity of depressive symptoms on the CES-D	20.48 (9.11)	19.60 (10.45)	.856^a^	21.75 (10.48)	15.73 (8.92)	<0.0001^a^
Level of QOL on the WHOQOL-BREF						
Physical health	22.93 (4.60)	23.27 (4.87)	.602^a^	21.47 (4.33)	24.05 (4.45)	<0.0001^a^
Mental health	16.11 (3.11)	17.73 (4.10)	.284^a^	16.89 (3.90)	18.88 (4.13)	<0.0001^a^
Social	12.86 (2.88)	12.97 (2.63)	.823^a^	12.03 (2.48)	12.83 (2.75)	<0.0001^a^
Environmental	27.43 (5.41)	29.10 (5.45)	.063^a^	27.15 (5.28)	29.39 (6.8)	<0.0001^a^

*Abbreviations:* *CES-D* Center for Epidemiological Studies Depression Scale, *MMT* methadone maintenance treatment, *QOL* quality of life, *SDS* ^*[Ch]*^ The Chinese version of the Severity of Dependence Scale, *WHOQOL-BREF* Taiwan brief version of the World Health Organization Quality of Life instrument. ^a^: paired *t*-test; ^b^: McNemar test.

Both groups reported heroin use at the intake interview. Rates of self-reported heroin use during the last month of the study were 66.67% and 61.32% for HIV-positive and HIV-negative participants, respectively. The urine-positive rates for the HIV and non-HIV groups were 73.33% and 66.51%, respectively. Both the rate of self-reported heroin use and the urine-positive rate did not differ between the two groups. The difference in methadone dosages (SD) was not significant, with 52.33 (20.03) (mg/day) and 57.56 (30.12) (mg/day) for the HIV and non-HIV groups, respectively.

Results of the comparison between treatment outcomes before and after MMT in heroin users with and without HIV infection are shown in Table 1. In participants without HIV infection, severity of heroin dependence, harm caused by heroin use and depressive symptoms and the proportion rate of current heroin use decreased significantly during the 3-month MMT; additionally, all four domains of QOL improved significantly during the 3-month MMT. In participants with HIV infection, the severity of heroin dependence and harm caused by heroin use were significantly improved after the 3-month MMT; the proportion rate of heroin use also significantly decreased. However, the severity of depressive symptoms and the levels of all four domains of QOL did not significantly improve in participants with HIV infection after 3-month MMT.

Results of the comparison of changes in heroin dependence, harm caused by heroin use, current heroin use, depressive symptoms and QOL during the 3-month MMT between heroin users with and without HIV infection using mixed-model analysis are shown in Tables 2 and 3. The results indicated that after controlling for sex, age, years of education and attendance rates, there were no significant differences in the changes in severity of heroin dependence and harm caused by heroin use and the proportion rate of current heroin use during the 3-month MMT between heroin users with and without HIV infection. However, heroin users without HIV infection showed greater improvement in the severity of depressive symptoms than those with HIV infection during 3-month MMT. Regarding QOL, heroin users without HIV infection had greater improvement in the physical health domain of QOL than those with HIV infection. There were no significant differences in the changes in other domains of QOL during 3-month MMT between heroin users with and without HIV infection.

**Table 2:** **Group comparisons of the changes in heroin dependence, harm caused by heroin use and current heroin use: mixed-model analysis**

	**Severity of heroin dependence**		**Severity of harm caused by heroin use**		**Current heroin use**	
	**Coefficient**	***p***	**Coefficient**	***p***	**OR (95% CI of OR)**	***p***
HIV positive serostatus	−0.371	.613	−2.531	.061	1.430 (0.517-3.952)	.489
Sex: male	−0.931	.221	0.225	.870	1.163 (0.431-3.134)	.765
Age	−0.01	.786	−0.181	.004	0.985 (0.947-1.025)	.468
Education level	−0.058	.625	−0.199	.307	1.090 (0.966-1.231)	.161
Attending rate	−5.050	.002	−2.120	.460	0.034 (0.002-0.428)	.013

*Abbreviations:* *CI* confidence interval, *OR* odds ratio.

**Table 3:** **Group comparisons of changes in depressive symptoms and quality of life: mixed-model analysis**

	**Depressive symptoms**		**Quality of life**							
			**physical health**		**psychological**		**social**		**environmental**	
	**Coefficient**	***p***	**Coefficient**	***p***	**Coefficient**	***p***	**Coefficient**	***p***	**Coefficient**	***p***
HIV positive serostatus	5.588	.005	−2.136	.016	−1.245	.110	−0.699	.172	−0.516	.615
Sex: male	−5.414	.009	1.150	.205	2.255	.005	0.999	.057	1.441	.171
Age	0.144	.122	−0.018	.657	0.007	.835	0.028	.232	−0.032	.504
Education	−0.055	.850	−0.003	.981	−0.004	.974	0.008	.931	0.038	.796
Attending rate	−1.952	.648	3.942	.038	2.221	.181	1.706	.118	2.385	.277

## Discussion

This study determined the changes in severity of heroin dependence, harm caused by heroin use, current heroin use, depressive level and QOL during 3-month MMT in heroin users with and without HIV infection. We observed that in heroin users both with and without HIV infection, three primary outcome indicators related to heroin use improved significantly after 3-month MMT, namely the severity of dependence and harm caused by heroin use and current heroin use. Two meta-analysis studies showed that MMT can reduce heroin use [30,31]. However, these studies did not analyze the effect of MMT on reducing heroin use in individuals who were infected with HIV. We further compared the changes in these three heroin use-related outcome indicators between heroin users with and without HIV infection and found that MMT has similar effects in both groups. Therefore, the results of this study support that MMT can reduce heroin use and the harm caused by heroin use, regardless of HIV serostatus.

Nevertheless, we found that the effects of MMT on improving depressive symptoms and QOL are different between heroin users with and without HIV infection. In contrast to heroin users who are negative for HIV, the severity of depressive symptoms did not significantly improve after 3-month MMT in the HIV-infected heroin users. Comparison of the changes in depressive symptoms also showed that heroin users without HIV infection had greater improvement in depressive symptoms than HIV-infected users. Tsao et al. showed that people with HIV infection experience more pain and distress than the general population [32]. Pain is not only a risk factor for developing depressive symptoms [33] but can also be a depressive symptom [34,35]. In addition, distress [36], stigma towards people with HIV [37], and a high virus load are associated with severity of depressive symptoms [38,39]. In addition, in the early stages of HIV infection, a reduced level of serotonin in the brain may be associated with the development of depression [40]. These findings of previous studies have supported that heroin users with HIV infection may be more vulnerable to the development of depressive symptoms than those without HIV infection, and have partially accounted for the difference in the effect of MMT on improving depressive symptoms between heroin users with and without HIV infection. The results of our study indicated that heroin users with HIV infection may need interventions in addition to MMT to improve their depressive symptoms. Pyne et al. reviewed previous studies on the care of patients with HIV infection and suggested that antidepressants may improve depressive symptoms in HIV-positive patients [41]. Psychological intervention, particularly intervention with a cognitive-behavioral component, has been shown to be effective in treating depressive symptoms among individuals with HIV infection who are drug users [15,42]. Research has also shown that depressive symptoms in HIV-infected individuals require multicomponent interventions [43]. Comprehensive treatments integrating pharmacological and psychosocial interventions may be essential to improve depressive symptoms in heroin users with HIV infection.

We found that heroin users without HIV infection also had greater improvement in the physical health domain of QOL than those with HIV infection after controlling for age, sex, education level, and attendance rate of MMT. Previous studies have found that HIV-positive individuals have low rates of coordinated and continuous health care use [44] but disproportionately high hospitalization rates [45]. Furthermore, health care utilization is lower for persons with HIV infection who use illicit drugs than for those who do not [46]. HIV-positive individuals reporting crack or cocaine use were also less likely to positively perceive quality of health care than those who were not substance abusers [47]. The results of this and previous studies indicate that the physical domain of QOL in HIV-infected heroin users requires integrated treatment programs, for example cooperation between professionals in the fields of addiction and infectious disease.

There are some limitations to this study that should be addressed. First, imbalance in the sample sizes of the groups of heroin users with and without HIV infection may limit the inferences of our findings. However, we used mixed-model analysis, an effective method in unbalanced samples, to deal with this problem [48]. Second, the rates of self-reported heroin use in this study were lower than the urine-positive rates in both groups. Therefore, current heroin use was determined based on self-reporting or a urine test to avoid underestimating heroin use; the literature has shown that self-reporting among drug users in research settings has acceptable reliability and validity [49,50]. Third, the follow-up period of this study was 3 months. Further study is needed to examine whether treatment outcomes differ between heroin users with and without HIV infection if the follow-up period is longer.

In conclusion, this study found that the primary outcomes regarding heroin use are improved during 3-month MMT in heroin users both with and without HIV infection, whereas poorer improvements in depressive symptoms and the physical domain of QOL were found in HIV-infected heroin users compared with those without HIV infection. Owing to the high prevalence rate of HIV-positive serostatus among heroin users, clinicians should take mood and QOL into consideration and incorporate multicomponent treatment programs when treating heroin users. Connock et al. showed that adjunctive psychosocial interventions may enhance the treatment outcomes of MMT [51]. Archana et al. suggested that simultaneous use of evidence-based substance use treatment and psychiatric care is needed to improve health outcomes in heroin users with HIV infection [52]. How to best help HIV-infected heroin users to improve their treatment outcomes, especially depressive symptoms and the physical domain of QOL, requires further study.
